# Prognostic Impact of Immunoscore in Pathological Stage III Differentiated Gastric Cancer: A Multicenter Cohort Study Including PD‐L1/PD‐L2 Expression Analysis

**DOI:** 10.1002/ags3.70114

**Published:** 2025-10-29

**Authors:** Yoshiro Yukawa, Takuro Saito, Yukinori Kurokawa, Yusuke Akamaru, Shinya Kidogami, Hiroshi Imamura, Kazumasa Fujitani, Jin Matsuyama, Kazuyoshi Yamamoto, Tsuyoshi Takahashi, Takahiro Matsui, Eiichi Morii, Hidetoshi Eguchi, Yuichiro Doki

**Affiliations:** ^1^ Department of Gastroenterological Surgery Osaka University Graduate School of Medicine Osaka Japan; ^2^ Department of Surgery Osaka Rosai Hospital Osaka Japan; ^3^ Department of Gastroenterological Surgery Saiseikai Senri Hospital Osaka Japan; ^4^ Department of Gastroenterological Surgery Toyonaka Municipal Hospital Osaka Japan; ^5^ Department of Gastroenterological Surgery Osaka General Medical Center Osaka Japan; ^6^ Department of Gastroenterological Surgery Higashiosaka City Medical Center Osaka Japan; ^7^ Department of Pathology Osaka University Graduate School of Medicine Osaka Japan

**Keywords:** gastric cancer, histological type, Immunoscore, PD‐L1, PD‐L2

## Abstract

**Background:**

The clinical use of the tumor microenvironment as a biomarker remains difficult in gastric cancer (GC). This multicenter, retrospective cohort study assessed the prognostic ability of the Immunoscore (IS) and the expression of programmed death ligand 1 (PD‐L1) or programmed death ligand 2 (PD‐L2) to select GC patients at higher risk of recurrence who may therefore require more intensive perioperative treatment.

**Methods:**

In 184 untreated pStage III GC patients who underwent radical gastrectomy at 13 institutions, IS (CD3+ and CD8+ lymphocytes) and PD‐L1/2 expression were analyzed by immunohistochemistry using digital pathology HALO software. The associations between clinicopathological factors and prognosis were assessed.

**Results:**

Neither IS nor PD‐L1/2 expression was a prognostic factor in the overall cohort. Subgroup analysis by histological type showed that in patients with differentiated‐type GC, the high IS group had significantly better recurrence‐free survival (RFS) (hazard ratio, 0.39; 95% confidence interval, 0.19–0.78; log‐rank *p* = 0.006) and overall survival (OS) (hazard ratio, 0.39; 95% confidence interval, 0.19–0.82; log‐rank *p* = 0.009) than the low IS group, whereas in undifferentiated‐type cases, IS was not associated with RFS or OS. Cox multivariate analysis revealed that IS was an independent prognostic factor for RFS (*p* = 0.024) and OS (*p* = 0.017) only in differentiated‐type cases.

**Conclusions:**

Histological classification may be useful in assessing the tumor microenvironment in GC, and patients with differentiated‐type pStage III with a low IS signature may be candidates for more intensive perioperative treatment due to their higher risk of recurrence.

## Graphical abstract

We assessed the prognostic ability of the Immunoscore and PD‐L1 or PD‐L2 expression in pStage III GC patients by immunohistochemistry. The results showed that Immunoscore was an independent prognostic factor in differentiated GC, but not in undifferentiated GC.

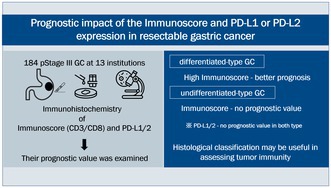

## 1 Introduction

Gastric cancer (GC) is the fifth leading cause of cancer‐related deaths worldwide [1], and has the highest incidence and mortality rate among all gastroenterological malignancies in Japan [2]. Surgery is the main treatment for operable GC, but patients with locoregional GC frequently develop tumor recurrence, and the prognosis for those with recurrent GC treated by conventional chemotherapy remains poor [3]. To reduce the recurrence rate after surgery, postoperative adjuvant chemotherapy is usually performed for pStage II or III GC patients [4]. However, the recurrence rate in patients with pStage III GC is still high after standard adjuvant chemotherapy; thus, it is necessary to develop new techniques to identify which of these patients have a particularly high risk of recurrence and may therefore be candidates for more intensive perioperative treatment.

The tumor microenvironment (TME), represented by tumor‐infiltrating lymphocytes (TILs), plays an important role in cancer progression and patient survival [5]. The Immunoscore (IS), a biomarker calculated on the basis of the densities of TILs, mainly CD3+ and CD8+ lymphocytes, has been reported to be associated with prognosis in colorectal cancer [6]. One retrospective study reported that TILs were a potential prognostic marker for GC [7]; however, because of different and subjective evaluation methods, studies evaluating the same CD8+ lymphocytes had strikingly different results [8, 9].

The programmed cell death 1 (PD‐1) pathway plays an important role in cancer progression in the TME [10]. PD‐1 downregulates the immune response by interacting with programmed death ligand 1 (PD‐L1) and programmed death ligand 2 (PD‐L2), and this enables cancer cells to escape immune destruction [11]. Regarding PD‐L1, the tumor proportion score (TPS) and a combined positive score quantifying PD‐L1 expression have been used as predictive biomarkers for immunotherapy in some cancers, including GC [12, 13], but the relationship between PD‐L1 expression and prognosis in GC remains controversial [14]. Regarding PD‐L2, its expression has been reported to be a potential predictive biomarker for immunotherapy in some cancers [15], but the relationship between PD‐L2 expression and prognosis in GC remains unclear [16].

Thus, the evaluation of the TME, as represented by IS and PD‐L1 or PD‐L2 (PD‐L1/2) expression, may provide important information on the biological and immunological characteristics of GC, and may serve as a comprehensive prognostic indicator independent of conventional TNM staging. Furthermore, most previous studies evaluating IS or PD‐L1/2 targeted unresectable or recurrent GC, rather than resectable GC. The present multicenter, retrospective cohort study assessed the prognostic ability of IS and PD‐L1/2 expression in resectable pStage III GC to select patients at high risk of recurrence for whom more intensive perioperative treatment is indicated.

## 2 Methods

### 2.1 Patients

This multicenter, retrospective cohort study included pStage III GC patients who underwent radical gastrectomy between January 2008 and December 2010 at 13 institutions as part of the Clinical Study Group of Osaka University, Upper Gastrointestinal Surgery Group. Exclusion criteria were as follows: synchronous or metachronous cancer within 5 years, and a history of preoperative treatment. Patients with mismatch repair deficiency were also excluded from this study because they have a favorable prognosis and demonstrate minimal benefit from perioperative chemotherapy [17]. TNM staging was performed according to the 7th edition of the Union for International Cancer Control TNM classification [18]. In principle, gastrectomy, lymph node dissection, adjuvant chemotherapy, and postoperative follow‐up were carried out according to the 3rd edition of the Japanese Gastric Cancer Treatment Guidelines [19]. All patients who received adjuvant chemotherapy were treated with S‐1, and none received immune checkpoint inhibitors during the perioperative period. Histological type classification was defined by the Japanese Classification of Gastric Carcinoma as follows: differentiated‐type GC consisted of papillary adenocarcinoma, well‐differentiated tubular adenocarcinoma, and moderately differentiated tubular adenocarcinoma, while undifferentiated‐type GC consisted of poorly differentiated adenocarcinoma, signet‐ring‐cell adenocarcinoma, and mucinous adenocarcinoma [19]. Within the stained slides, those with a high proportion of differentiated components were classified as differentiated‐type, and those with a high proportion of undifferentiated components were classified as undifferentiated‐type. This study was approved by the institutional review board of Osaka University Hospital, number 21312. This study was registered with UMIN Clinical Trials Registry, number 000046593.

### 2.2 Immunohistochemistry

Tumor tissue samples were obtained from surgically resected specimens that were fixed in 10% neutral buffered formalin for 24–72 h in most cases, then embedded in paraffin. Pathologists at each institution, who were unaware of the clinical data, selected all the formalin‐fixed paraffin‐embedded (FFPE) tissues containing the deepest part of the tumor, and these samples were shipped to the study center. All FFPE samples were cut into 4‐μm sections for immunohistochemical (IHC) staining performed using the Dako Autostainer Link 48+ (Agilent Technologies, Santa Clara, CA, USA), with incubation with primary antibodies for 30 min at room temperature according to the manufacturer's instructions. The specificities of the monoclonal antibodies used in IHC staining of FFPE samples were confirmed with human tonsil tissue sections (positive control). Every stained slide was scanned with a Ventana iScan HT (Roche Diagnostics, Sant Cugat, Spain) to obtain 20× digital images. In all slides, the tumor core was independently defined by two observers (Y.Y. and T.S.) who were blinded to the clinicopathological data, with disagreements resolved by consensus. Whole‐slide images were analyzed by digital pathology using HALO software (Indica Labs, Corrales, NM, USA) to identify and quantify stained cells. HALO software enables automated quantification of immunostaining, providing high reproducibility and efficiency, though its accuracy can be influenced by slide quality and staining variability, and proper parameter optimization is necessary.

### 2.3 Immunoscore Evaluation

**FIGURE 1:**
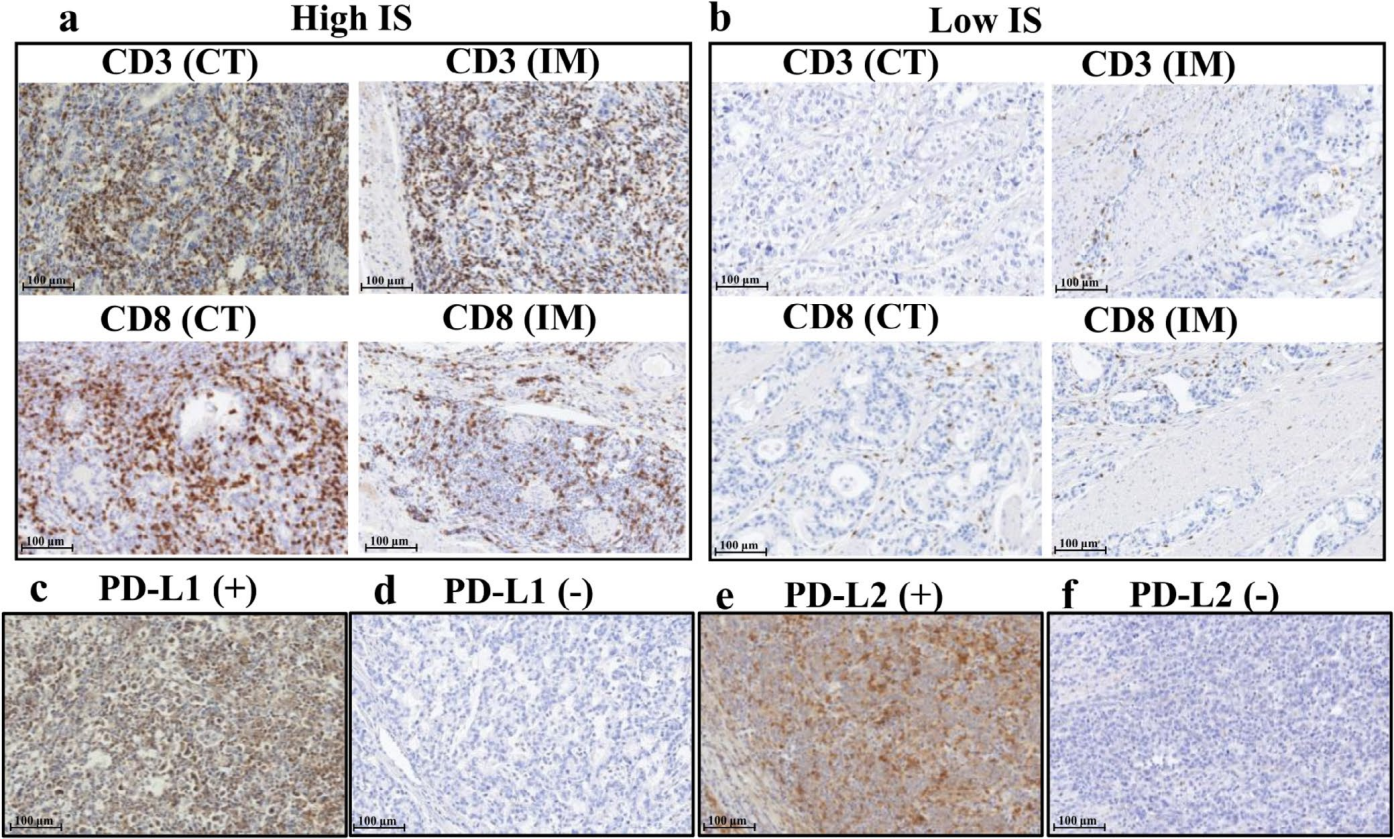
Representative images of (a) high Immunoscore, (b) low Immunoscore, (c) PD‐L1 (+), (d) PD‐L1 (−), (e) PD‐L2 (+), and (f) PD‐L2 (−).

IS was evaluated by IHC staining of CD3+ and CD8+ lymphocytes. The primary antibodies were monoclonal antibodies for CD3 (SP7, 1:200; Abcam, Cambridge, UK) and CD8 (C8/144B, 1:100; Agilent Technologies) as previously reported [6]. IS was calculated on the basis of a recent large cohort of colorectal cancer, which quantified the densities of CD3+ and CD8+ lymphocytes in the core of the tumor (CT) and in the invasive margin (IM) [6]. In brief, the IM region was defined as the area 500 μm from the inside and outside of the boundary between normal tissue and tumor tissue, and the CT region was defined as all tumor areas within the IM region. The percentiles of CD3+ and CD8+ lymphocytes in the CT and IM areas were determined in each specimen, and the averages of these four percentile scores were calculated. The low IS and high IS groups were classified on the basis of mean percentiles of 0%–25% and 25%–100%, respectively, according to the previous study [6]. Representative IHC images with high or low IS are shown in Figure 1a,b, respectively.

### 2.4 Evaluation of PD‐L1 and PD‐L2 Expression

IHC staining for PD‐L1 was performed using PD‐L1 IHC 28‐8 pharmDx DAKO (Agilent Technologies) as previously reported [20, 21]. IHC staining for PD‐L2 was performed using monoclonal antibodies against PD‐L2 (EPR25200‐50, 1:1600; Abcam) as the primary antibody. PD‐L1/2 expression was defined as positive when tumor cell membranes were stained in comparison with those of the positive control. For the evaluation of PD‐L1/2 expression, the TPS was defined as the number of PD‐L1–stained tumor cells divided by the total number of viable tumor cells multiplied by 100. In this study, the cut‐off value for the TPS of PD‐L1 was set at 1 according to the results of recent clinical trials [20, 21]. The cut‐off value for the TPS of PD‐L2 was also set at 1, according to the findings of a different previous study [22]. Representative IHC images with PD‐L1/2 expression are shown in Figure 1c–f.

### 2.5 Statistical Analysis

Associations between clinicopathological factors were compared using the chi‐square test for categorical variables and the Mann–Whitney *U* test for continuous variables. Recurrence‐free survival (RFS) was defined as the time from surgery to either the first recurrence or death from any cause. Overall survival (OS) was defined as the time from surgery to death from any cause. RFS and OS curves were estimated using the Kaplan–Meier method, and survival differences were compared using the log‐rank test. To evaluate independent prognostic significance, Cox proportional hazards models were used for both univariate and multivariate analyses. A *p* value < 0.05 was considered to be statistically significant. All statistical calculations were performed using JMP version 14 software (SAS Institute, Cary, NC, USA) or SPSS Statistics version 24 (IBM Corp, Armonk, NY, USA).

## 3 Results

### 3.1 Clinicopathological Characteristics

This study included a total of 184 GC patients. Clinicopathological characteristics according to IS and PD‐L1/2 expression are shown in Table 1. The proportion of patients with high IS, PD‐L1 (+), and PD‐L2 (+) were 80%, 18%, and 35%, respectively. There were no background factors, including histological type, that were statistically associated with IS or PD‐L1/L2 status. IS was significantly higher in the PD‐L1 (+) group than in the PD‐L1 (−) group, while PD‐L2 expression was not associated with IS status. In terms of the correlation between PD‐L1 and PD‐L2 expression, 9% of patients were double positive, 57% were double negative, and the proportion who were PD‐L2 (+) was significantly higher in the PD‐L1 (+) group than in the PD‐L1 (−) group (*p* = 0.013).

**TABLE 1:** Clinicopathological characteristics according to the Immunoscore (IS), PD‐L1 expression, and PD‐L2 expression.

	High IS (*n* = 147)	Low IS (*n* = 37)	*P*	PD‐L1 (+) (*n* = 31)	PD‐L1 (−) (*n* = 153)	*p*	PD‐L2 (+) (*n* = 65)	PD‐L2 (−) (*n* = 119)	*p*
Age									
Median (range)	71 (34–89)	73 (45–90)	0.67	74 (36–88)	70 (34–90)	0.13	70 (44–88)	72 (34–90)	0.97
Sex									
Male	106 (72%)	25 (68%)	0.59	20 (65%)	111 (73%)	0.37	48 (74%)	83 (70%)	0.56
Female	41 (28%)	12 (32%)		11 (35%)	42 (27%)		17 (26%)	36 (30%)	
Location									
Upper	32 (22%)	7 (19%)	0.71	8 (26%)	31 (20%)	0.49	10 (15%)	29 (24%)	0.15
Middle or lower	115 (78%)	30 (81%)		23 (74%)	122 (80%)		55 (85%)	90 (76%)	
Histological type									
Differentiated	54 (37%)	15 (41%)	0.67	8 (26%)	61 (40%)	0.14	24 (37%)	45 (38%)	0.91
Undifferentiated	93 (63%)	22 (59%)		23 (74%)	92 (60%)		41 (63%)	74 (62%)	
pT									
2, 3	49 (33%)	14 (38%)	0.61	9 (29%)	54 (35%)	0.50	26 (40%)	37 (31%)	0.22
4	98 (67%)	23 (62%)		22 (71%)	99 (65%)		39 (60%)	82 (69%)	
pN									
0, 1	32 (22%)	4 (11%)	0.13	10 (32%)	26 (17%)	0.051	9 (14%)	27 (23%)	0.15
2, 3	115 (78%)	33 (89%)		21 (68%)	127 (83%)		56 (86%)	92 (77%)	
Adjuvant chemotherapy									
Yes	91 (62%)	22 (59%)	0.78	21 (68%)	92 (60%)	0.43	44 (68%)	69 (58%)	0.20
No	56 (38%)	15 (41%)		10 (32%)	61 (40%)		21 (32%)	50 (42%)	
IS									
High				30 (97%)	117 (76%)	0.010	54 (83%)	93 (78%)	0.43
Low				1 (3%)	36 (24%)		11 (17%)	26 (22%)	
PD‐L1 expression									
(+)	30 (20%)	1 (3%)	0.010				17 (26%)	14 (12%)	0.013
(−)	117 (80%)	36 (97%)					48 (74%)	105 (88%)	
PD‐L2 expression									
(+)	54 (37%)	11 (30%)	0.43	17 (55%)	48 (31%)	0.013			
(−)	93 (63%)	26 (70%)		14 (45%)	105 (69%)				

Abbreviations: IS, Immunoscore; PD‐L1, programmed death‐ligand 1; PD‐L2, programmed death‐ligand 2.

### 3.2 Kaplan–Meier Survival Analysis

**FIGURE 2:**
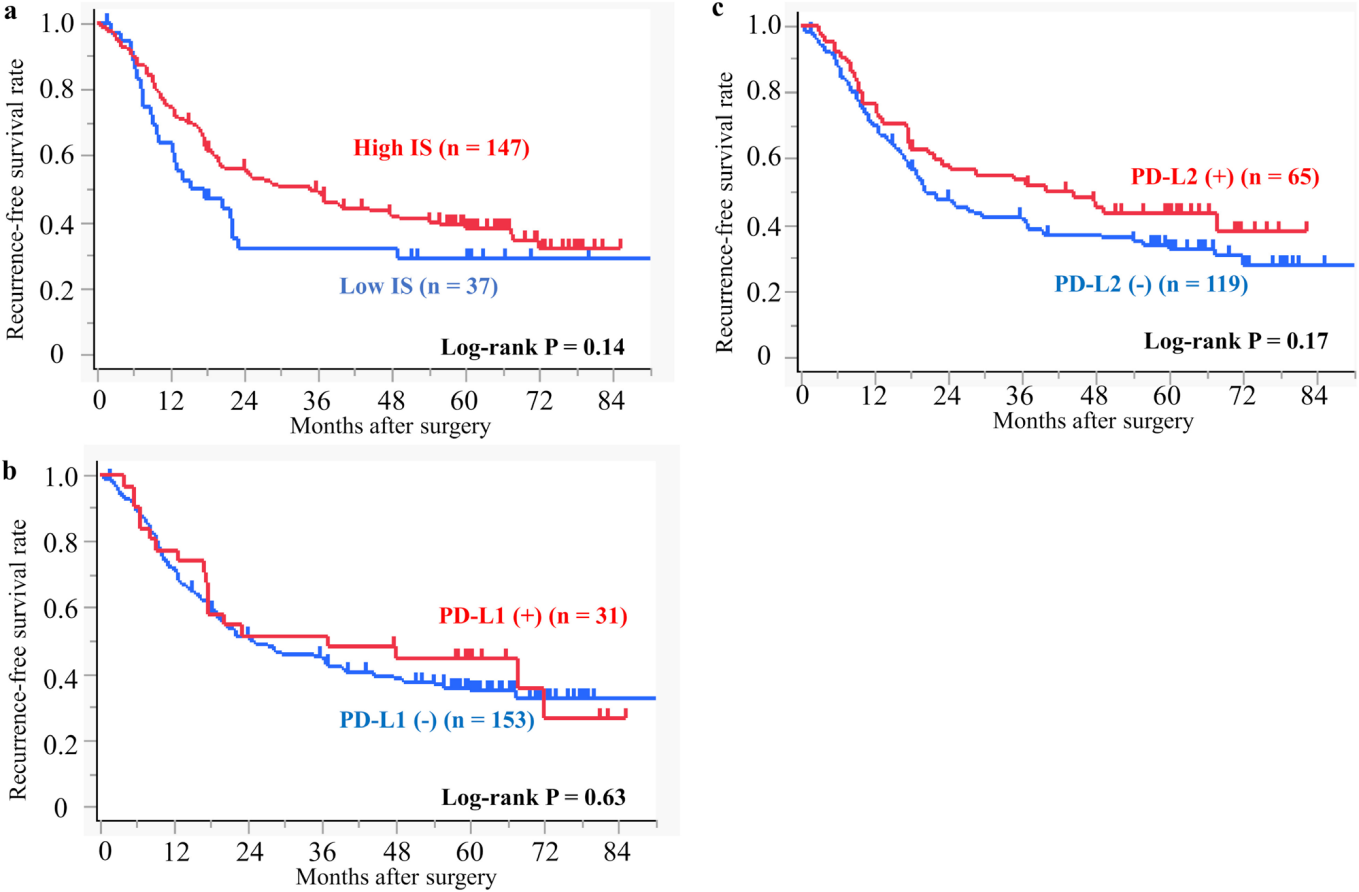
Kaplan–Meier survival curves of recurrence‐free survival according to (a) Immunoscore, (b) PD‐L1 expression, and (c) PD‐L2 expression. The significance of differences was calculated using the log‐rank test.

In the survival analysis for RFS, Kaplan–Meier curves showed a separation between the high IS group and the low IS group, but the difference was not statistically significant (hazard ratio [HR], 0.72; 95% confidence interval [CI], 0.46–1.12; log‐rank *p* = 0.14) (Figure 2a). On the other hand, there was a relatively small difference in RFS between the PD‐L1 (+) and PD‐L1 (−) groups (HR, 0.88; 95% CI, 0.54–1.45; log‐rank *p* = 0.63) (Figure 2b) and between the PD‐L2 (+) and PD‐L2 (−) groups (HR, 0.76; 95% CI, 0.51–1.12; log‐rank *p* = 0.17) (Figure 2c). The survival analysis for OS showed a similar pattern as that seen with RFS. The difference in OS between the high and low IS groups (HR, 0.69; 95% CI, 0.43–1.12; log‐rank *p* = 0.13) was larger than that between the PD‐L1 (+) and PD‐L1 (−) groups (HR, 0.92; 95% CI, 0.54–1.58; log‐rank *p* = 0.77) and also the PD‐L2 (+) and PD‐L2 (−) groups (HR, 0.87; 95% CI, 0.57–1.31; log‐rank *p* = 0.50) (Figure S1a–c).

### 3.3 Subgroup Analyses According to Histological Type

**FIGURE 3:**
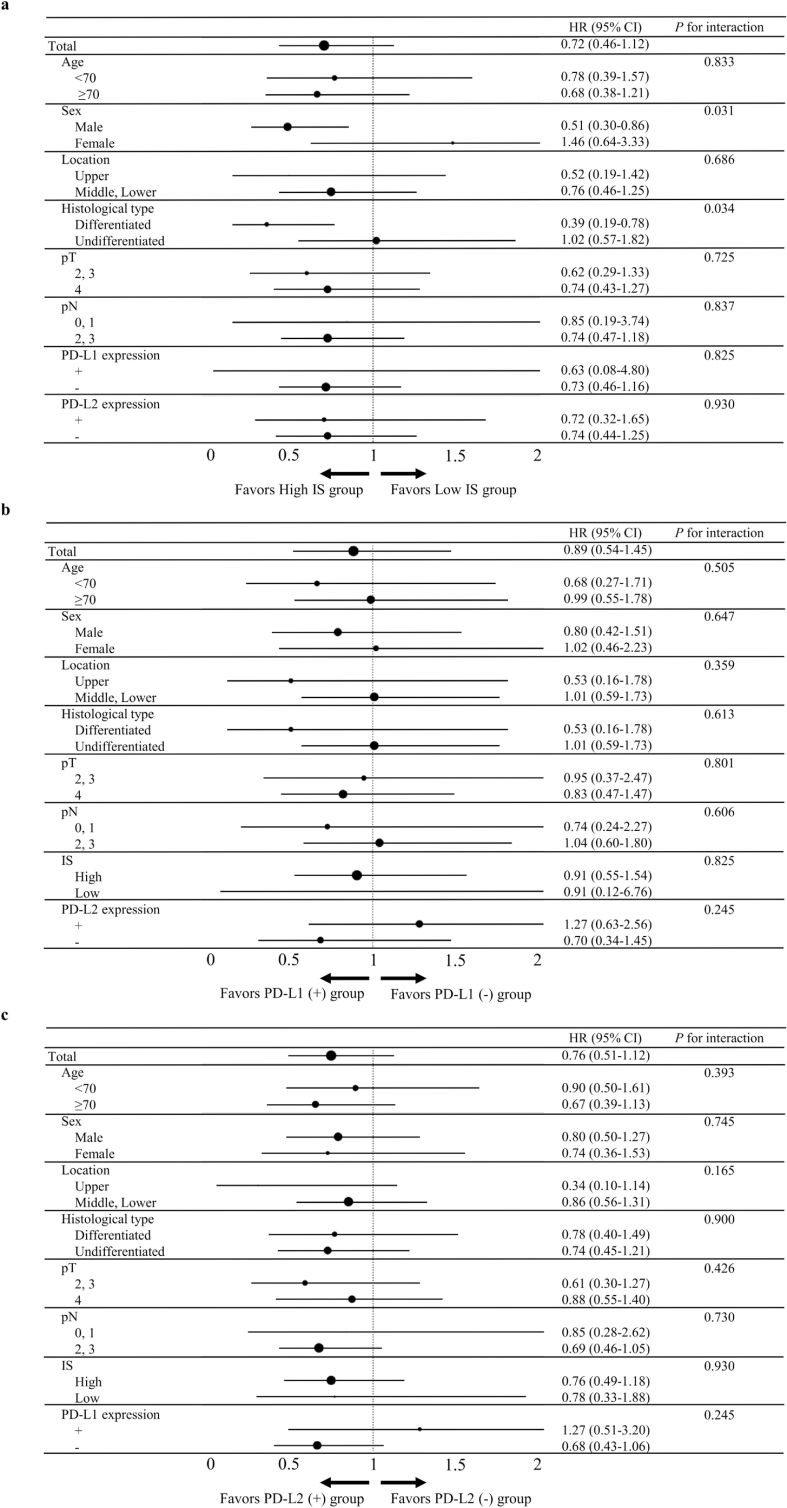
Forest plot of the impact of (a) Immunoscore, (b) PD‐L1 expression, and (c) PD‐L2 expression on recurrence‐free survival according to patient subgroup.

In a subgroup analysis of RFS, IS showed a significant interaction with sex (*p* = 0.031) and histological type (*p* = 0.034) (Figure 3a), while neither PD‐L1 nor PD‐L2 expression showed a significant interaction with any factor (Figure 3b,c). A similar pattern was observed in a subgroup analysis of OS (Figure S2a–c). When we examined the heterogeneity of background characteristics between differentiated‐ and undifferentiated‐type GC, the percentage of female patients was significantly higher for the undifferentiated type (*p* < 0.001) (Table S1).

**FIGURE 4:**
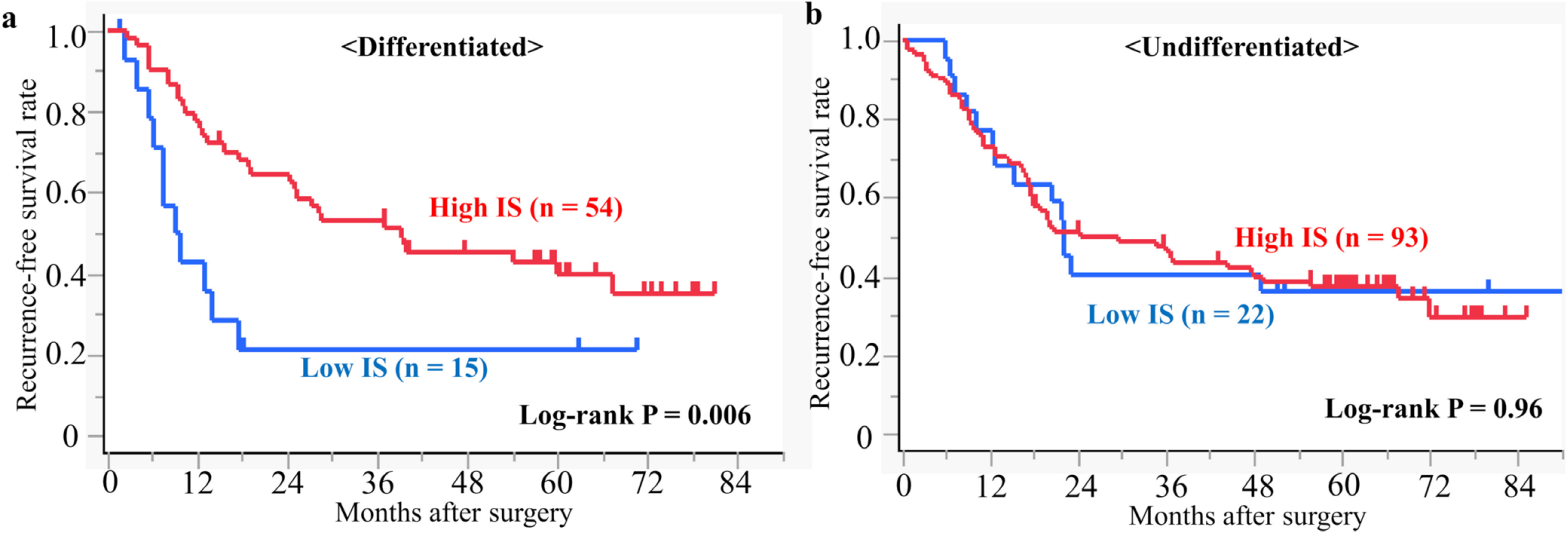
Kaplan–Meier survival curves of recurrence‐free survival in (a) differentiated‐type and (b) undifferentiated‐type cases according to IS. The significance of differences was calculated using the log‐rank test.

We therefore compared the clinicopathological characteristics between the high and low IS groups after subgrouping according to histological type (Table 2). The high IS group contained a significantly higher percentage of PD‐L1 (+) patients than the low IS group, both for the differentiated‐type (*p* = 0.040) and undifferentiated‐type (*p* = 0.023) subgroups. In the RFS analysis, the high IS group showed significantly better RFS than the low IS group in the differentiated‐type subgroup (HR, 0.39; 95% CI, 0.19–0.78; log‐rank *p* = 0.006) (Figure 4a), but RFS was similar between the two IS groups in the undifferentiated‐type subgroup (HR, 1.02; 95% CI, 0.57–1.82; log‐rank *p* = 0.96) (Figure 4b). Similarly, in the OS analysis, the high IS group showed significantly better OS than the low IS group in the differentiated‐type subgroup (HR, 0.39; 95% CI, 0.19–0.82; log‐rank *p* = 0.009) (Figure S3a), whereas OS was similar between the two IS groups in the undifferentiated‐type subgroup (HR, 0.96; 95% CI, 0.50–1.84; log‐rank *p* = 0.90) (Figure S3b). Furthermore, we evaluated the association between the combination of IS and PD‐L1/2 and prognosis by histological classification. In the differentiated‐type subgroup, low IS was a poor prognostic factor for RFS, but the combination analysis did not reveal a new subgroup with favorable or unfavorable prognosis (Figure S4a,c). In the undifferentiated‐type subgroup, there were no significant differences in RFS between any of the combinations (Figure S4b,d).

**TABLE 2:** Clinicopathological characteristics according to the Immunoscore (IS) in differentiated‐type and undifferentiated‐type cases.

	Differentiated			Undifferentiated		
	High IS (*n* = 54)	Low IS (*n* = 15)	*p*	High IS (*n* = 93)	Low IS (*n* = 22)	*p*
Age						
Median (range)	74 (34–89)	76 (57–89)	0.79	70 (36–89)	72 (45–90)	0.74
Sex						
Male	48 (89%)	11 (73%)	0.13	58 (62%)	14 (64%)	0.91
Female	6 (11%)	4 (27%)		35 (38%)	8 (36%)	
Location						
Upper	12 (22%)	2 (13%)	0.45	20 (22%)	5 (23%)	0.90
Middle or lower	42 (78%)	13 (87%)		73 (78%)	17 (77%)	
pT						
2, 3	24 (44%)	7 (47%)	0.88	25 (27%)	7 (32%)	0.64
4	30 (56%)	8 (53%)		68 (73%)	15 (68%)	
pN						
0, 1	11 (20%)	1 (7%)	0.22	21 (23%)	3 (14%)	0.35
2, 3	43 (80%)	14 (93%)		72 (77%)	19 (86%)	
Adjuvant chemotherapy						
Yes	35 (65%)	7 (47%)	0.20	56 (60%)	15 (68%)	0.49
No	19 (35%)	8 (53%)		37 (40%)	7 (32%)	
PD‐L1 expression						
(+)	8 (15%)	0 (0%)	0.040	22 (24%)	1 (5%)	0.023
(−)	46 (85%)	15 (100%)		71 (76%)	21 (95%)	
PD‐L2 expression						
(+)	21 (39%)	3 (20%)	0.17	33 (35%)	8 (36%)	0.94
(−)	33 (61%)	12 (80%)		60 (65%)	14 (64%)	

Abbreviations: IS, Immunoscore; PD‐L1, programmed death‐ligand 1; PD‐L2, programmed death‐ligand 2.

Finally, to assess the prognostic value of IS independently of other confounding factors, we conducted Cox univariate and multivariate analyses after subgrouping according to histological type. Cox multivariate analysis of RFS that included the three clinicopathological covariables with a *p* value less than 0.1 in the univariate analysis revealed that for the differentiated‐type subgroup, low IS (*p* = 0.024) and lack of adjuvant chemotherapy (*p* = 0.022) were independent indicators of poor RFS (Table 3a). Cox multivariate analysis for OS that included the two clinicopathological covariables with a *p* value less than 0.1 revealed that in the differentiated‐type subgroup, low IS was an independent indicator of poor OS (*p* = 0.017) (Table S2a). In the undifferentiated‐type subgroup, Cox multivariate analyses revealed that pT4 (*p* = 0.022), pN2–3 (*p* = 0.004), and lack of adjuvant chemotherapy (*p* < 0.001) were independent indicators of poor RFS (Table 3b), and lack of adjuvant chemotherapy (*p* < 0.001) was an independent indicator of poor OS (Table S2b).

**TABLE 3:** Univariate and multivariate analyses of recurrence‐free survival in (a) differentiated‐type and (b) undifferentiated‐type cases.

(a)					
Variables	Category	Univariate analysis		Multivariate analysis	
		HR (95% CI)	*p*	HR (95% CI)	*p*
Age (years)	≥ 70	1.52 (0.79–2.91)	0.21		
Sex	Female	1.95 (0.90–4.22)	0.092	0.80 (0.30–2.14)	0.66
Location	M/L	1.36 (0.63–2.94)	0.43		
pT	4	1.66 (0.90–3.07)	0.11		
pN	2, 3	1.85 (0.72–4.70)	0.20		
Adjuvant chemotherapy	No	2.24 (1.23–4.08)	0.009	2.21 (1.12–4.36)	0.022
IS	Low	2.58 (1.29–5.20)	0.008	2.50 (1.13–5.56)	0.024
PD‐L1 expression	(−)	1.40 (0.50–3.92)	0.52		
PD‐L2 expression	(−)	1.29 (0.67–2.47)	0.45		

(b)					
Variables	Category	Univariate analysis		Multivariate analysis	
		HR (95% CI)	*p*	HR (95% CI)	*p*
Age (years)	≥ 70	1.11 (0.70–1.76)	0.66		
Sex	Female	1.19 (0.74–1.90)	0.47		
Location	M/L	1.11 (0.63–1.97)	0.71		
pT	4	1.71 (0.98–2.98)	0.058	2.33 (1.31–4.14)	0.004
pN	2, 3	1.73 (0.93–3.22)	0.084	2.60 (1.36–4.96)	0.004
Adjuvant chemotherapy	No	2.83 (1.77–4.51)	< 0.001	3.09 (1.93–4.97)	< 0.001
IS	High	1.02 (0.57–1.82)	0.96		
PD‐L1 expression	(−)	1.05 (0.59–1.85)	0.87		
PD‐L2 expression	(−)	1.35 (0.83–2.20)	0.23		

Abbreviations: CI, confidence interval; HR, hazard ratio; IS, Immunoscore; M/L, middle or lower third of the stomach; PD‐L1, programmed death‐ligand 1; PD‐L2, programmed death‐ligand 2.

## 4 Discussion

In this multicenter, retrospective cohort study, we evaluated the association between IS or PD‐L1/2 expression and prognosis in resectable GC. IHC staining of surgical specimens showed that neither IS nor PD‐L1/2 expression was associated with prognosis in the overall cohort. However, in subgroup analyses by histological type, we found that low IS was associated with a poor prognosis in differentiated‐type cases, whereas there was no association between IS and prognosis in undifferentiated‐type cases. In addition, a Cox multivariate analysis showed that low IS was an independent poor prognostic factor only in differentiated‐type GC.

IS was initially evaluated in 2006 using CD3+ and CD45RO+ lymphocytes [23]; however, in a recent large study of colorectal cancer, IS was calculated using CD3+ and CD8+ lymphocytes and was reported to be a prognostic factor beyond TNM classification [6]. Our results suggest that IS calculated using only CD3+ and CD8+ lymphocytes may not be a prognostic factor for all histological GC subtypes. In contrast to previous studies of TILs in GC, our study ensured objectivity and reproducibility by analyzing the entire tumor area on each slide using HALO software. A previous study incorporating many immunological factors reported that assessing five of them (CD3 in IM, CD3 in CT, CD8 in IM, CD45RO in CT, and CD66b in IM) could effectively predict recurrence and survival in patients with GC [24]. Another study reported that immune cells such as regulatory T lymphocytes and M1/M2 macrophages had a significant impact on the prognosis of GC [5]. These reports suggest that various immune cells, not just CD3+ and CD8+ lymphocytes, should be evaluated in order to use IS as a prognostic biomarker that applies to all histological GC subtypes.

In the present study, subgroup analyses by histological type showed that IS calculated using only CD3+ and CD8+ lymphocytes was associated with prognosis in differentiated‐type cases. This result is consistent with a previous report on IS evaluation in colorectal cancer [6]; it is reasonable because highly and moderately differentiated adenocarcinomas account for nearly 90% of all colorectal cancers [25]. On the other hand, in undifferentiated‐type cases, IS calculated using only CD3+ and CD8+ lymphocytes was not associated with prognosis. Sugano et al. divided GC into differentiated and undifferentiated types, which generally correspond to intestinal and diffuse types, respectively, as defined by Lauren's classification [26, 27]. A previous study showed that effector molecules on intratumor CD8+ lymphocytes, such as granzyme B, perforin, and interferon gamma, were reduced in diffuse‐type GC [28]. In clinical trials, diffuse‐type GC showed worse survival rates following PD‐1 blockade [20, 21]. Although we did not evaluate the function of CD8+ lymphocytes in this study, the histological type didn't associate with the IS which quantified the density, but not the function, of the lymphocytes; therefore, we speculate that IS was not a prognostic factor for undifferentiated‐type GC because some CD8+ lymphocytes exhibit dysfunction that results in reduced anti‐tumor immunity. In differentiated gastric cancer, IS may be useful as a prognostic factor and possibly for predicting the response to immunotherapy.

There have been inconsistent findings between studies regarding whether or not PD‐L1 expression is associated with prognosis in GC, and these discrepancies may be due to differences in tumor staging, tumor heterogeneity, evaluation methods, treatment choices, and race. A recent meta‐analysis of 2298 patients from 11 studies showed no significant association between PD‐L1 expression and OS [14], which is consistent with our results. There are two general mechanisms of PD‐L1 expression in tumor cells: innate immune resistance and adaptive immune resistance [11]. In innate immune resistance, constitutive oncogenic signaling induces PD‐L1 expression in tumor cells, whereas in adaptive immune resistance, TILs induce PD‐L1 upregulation in these cells. As PD‐L1 suppresses excessive immune responses in non‐cancer patients, its expression reflects the activation of anti‐tumor immunity in cancer patients. In fact, our study showed that the PD‐L1 (+) group had significantly higher IS than the PD‐L1 (−) group, indicating elevated CD3+ and CD8+ lymphocytes in the former group. We hypothesize that PD‐L1 expression is an indirect marker that reflects adaptive immune resistance in response to engaged CD3+ and CD8+ lymphocytes, and represents a mechanism of negative feedback on immune activation rather than an absolute marker. As a result, PD‐L1 alone was not a reliable prognostic factor for GC in our study.

In contrast to PD‐L1, data on PD‐L2 expression and its prognostic value in GC are sparse. PD‐1 blockade was associated with a favorable clinical course in patients with PD‐L1 (+) tumors, and showed clinical efficacy in several types of cancers, including GC, in patients with PD‐L1 (−) tumors [21, 29]. Furthermore, Nakayama et al. suggested that PD‐L2 blockade enhanced the anti‐tumor activity of cytotoxic T lymphocytes against GC cells expressing PD‐L2 [22]. These findings indicate the immunosuppressive role of PD‐L2 in GC, but its role as a prognostic biomarker has not yet been elucidated. Our study is the first to examine the association between PD‐L2 expression and prognosis using IHC staining and automated cell count–based scoring in resected specimens of GC patients at multiple centers. Considering its immunosuppressive role, PD‐L2 expression was expected to be a poor prognostic factor in GC, as was shown to be the case in esophageal cancer [30], but this was not demonstrated in our study. This discrepancy suggests that the effects of PD‐L2 on immune suppression may differ between cancer types. In our study, PD‐L2 expression was not associated with IS status, but compared with the PD‐L2 (−) group, the PD‐L2 (+) group contained a significantly higher percentage of patients who were PD‐L1 (+). According to these results, we speculate that although PD‐L2 does not reflect adaptive immune resistance against CD3+ or CD8+ lymphocytes to the same degree as PD‐L1, it may be affected to a certain extent by immune activation in concert with PD‐L1. Further studies are required to examine the mechanisms of the PD‐1 pathway, including the coordination of PD‐L1 and PD‐L2 by specific cancer types and histological types.

We also examined the relationship between IS, PD‐L1/2 expression, and prognosis, but no subgroup with a favorable prognosis was identified other than IS in differentiated‐type GC. Accordingly, we did not observe an immunologically “hot” or “cold” subpopulation based on this combination. Further studies are warranted to investigate the correlation between immunological activity and the effect of PD‐1 blockade in cohorts of patients treated with PD‐1 inhibitors.

This study has several limitations. First, its aim was to identify high‐risk GC patients who require more intensive perioperative treatment than the standard postoperative chemotherapy; thus, we selected only pStage III GC cases. If we seek to examine the prognostic values of IS, PD‐L1, and PD‐L2 in the entire population of patients with resectable GC, further studies must include patients with pStage I–II GC cases. Second, IHC staining evaluation was based on only one slide per tumor containing the deepest area of each specimen; thus, not all intra‐tumor heterogeneity was considered. However, heterogeneity within the same slide was taken into account by using HALO software to evaluate the whole slide. Third, the optimal cut‐off values of IS and PD‐L1/2 expression remain unclear. Although we used the most frequently used cut‐off values for both IS and PD‐L1/2 expression in this study, additional research to determine reliable cut‐off values is needed to validate our results. Fourth, because endoscopic biopsy specimens may be more suitable than surgical specimens for evaluating preoperative treatment, future studies should assess the TME using biopsy specimens.

In conclusion, our results indicated that neither IS nor PD‐L1/2 expression was a prognostic factor in the absence of histological subgrouping. However, we found a significant association between IS and prognosis in differentiated‐type cases, which suggests the need for TME evaluation, especially when using IS, after subgrouping by histological type. Differentiated‐type pStage III cases with a low IS signature may be candidates for more intensive perioperative treatment due to the higher risk of recurrence. Uncovering the immune heterogeneity in GC by histological assessment may contribute to the establishment of personalized immunotherapy, which could ultimately improve survival in GC patients.

## Author Contributions

**Yoshiro Yukawa:** conceptualization, investigation, writing – original draft, validation, formal analysis, methodology, software, data curation, writing – review and editing. **Takuro Saito:** writing – review and editing, conceptualization, investigation, methodology, validation, formal analysis, data curation, software. **Yukinori Kurokawa:** writing – review and editing, supervision, conceptualization, methodology, data curation. **Yusuke Akamaru:** data curation, investigation, resources. **Shinya Kidogami:** data curation, investigation, resources. **Hiroshi Imamura:** investigation, resources, data curation. **Kazumasa Fujitani:** data curation, resources, investigation. **Jin Matsuyama:** investigation, data curation, resources. **Kazuyoshi Yamamoto:** conceptualization, investigation. **Tsuyoshi Takahashi:** conceptualization, investigation. **Takahiro Matsui:** conceptualization, methodology, investigation, validation, data curation, software, formal analysis. **Eiichi Morii:** conceptualization, validation, methodology, software, formal analysis, data curation. **Hidetoshi Eguchi:** supervision. **Yuichiro Doki:** supervision.

## Ethics Statement

All procedures were in accordance with the Helsinki Declaration. Ethical Review Board Osaka University Hospital approved the protocol for this study (approved no. 21312). Consent to participate was not considered necessarily.

## Conflicts of Interest

Y. Kurokawa is an Associate Editor of *Annals of Gastroenterological Surgery*. Y. Doki. is an Editorial Board member of *Annals of Gastroenterological Surgery*. The other authors have no relevant financial or non‐financial interests to disclose.

## Supporting information


**Figure S1:** Kaplan–Meier survival curves of overall survival according to (a) Immunoscore, (b) PD‐L1 expression, and (c) PD‐L2 expression. The significance of differences was calculated using the log‐rank test.

**Figure S2:** Forest plot of the impact of (a) Immunoscore, (b) PD‐L1 expression, and (c) PD‐L2 expression on overall survival according to patient subgroup.

**Figure S3:** Kaplan–Meier survival curves of overall survival in (a) differentiated‐type and (b) undifferentiated‐type cases according to IS. The significance of differences was calculated using the log‐rank test.

**Figure S4:** Kaplan–Meier curves of recurrence‐free according to the combination of Immunoscore (IS) and PD‐L1 expression in (a) differentiated‐type and (b) undifferentiated‐type cases and the combination of IS and PD‐L2 expression in (c) differentiated‐type and (d) undifferentiated‐type cases. The significance of differences was calculated using the log‐rank test.

**Table S1:** ags370114‐sup‐0005‐TableS1.docx.
