# Supplementary figures and images for: Prognostic Impact of Immunoscore in Pathological Stage III Differentiated Gastric Cancer: A Multicenter Cohort Study Including PD‐L1/PD‐L2 Expression Analysis
Source: Ann Gastroenterol Surg. 2025 Oct 29;10(2):431–42. doi: 10.1002/ags3.70114 (PMC12962046; doi:10.1002/ags3.70114)

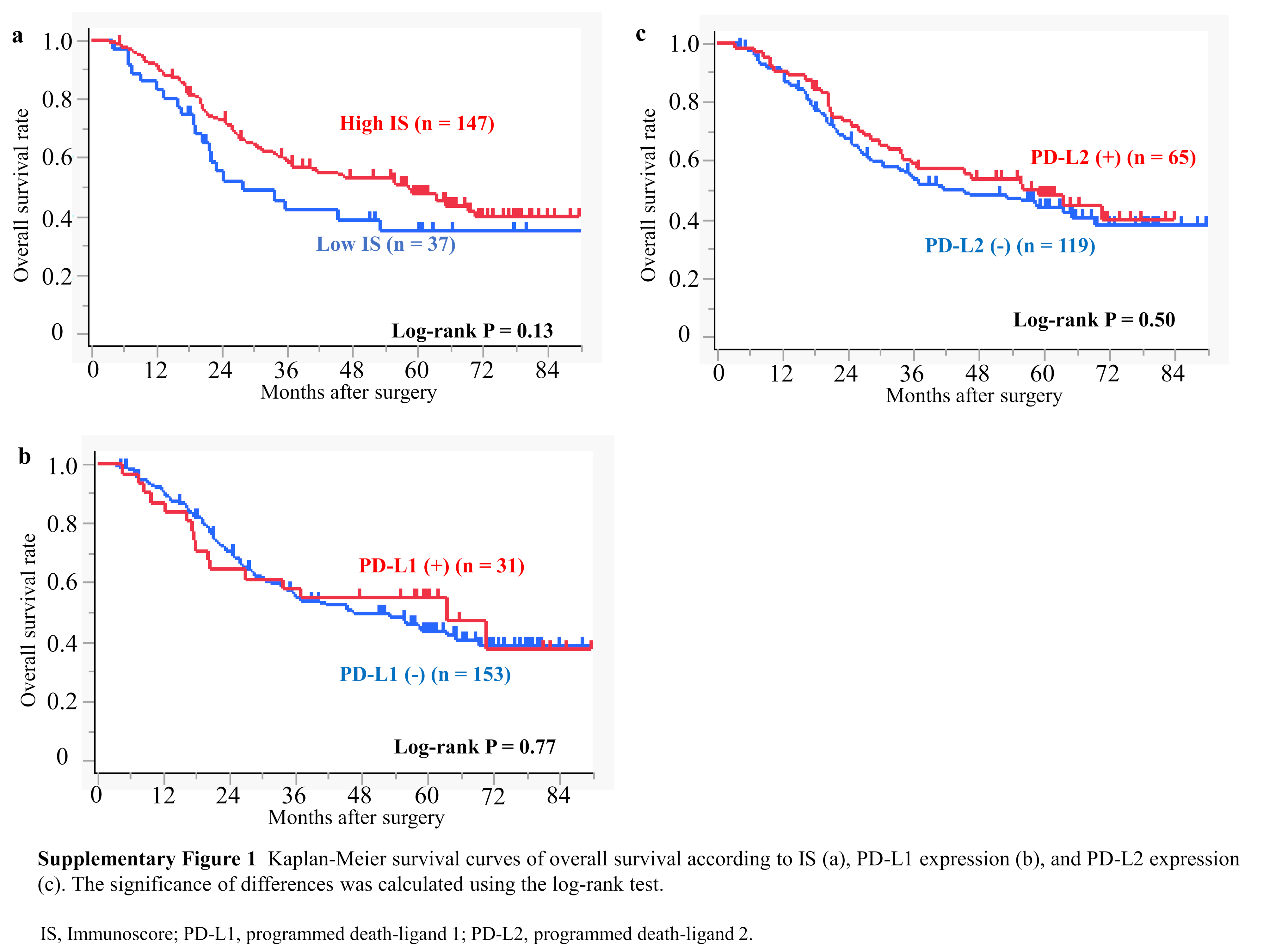

Supplement: Supplementary file 1 — Figure S1: Kaplan–Meier survival curves of overall survival according to (a) Immunoscore, (b) PD‐L1 expression, and (c) PD‐L2 expression. The significance of differences was calculated using the log‐rank test. [file AGS3-10-431-s004.tif]

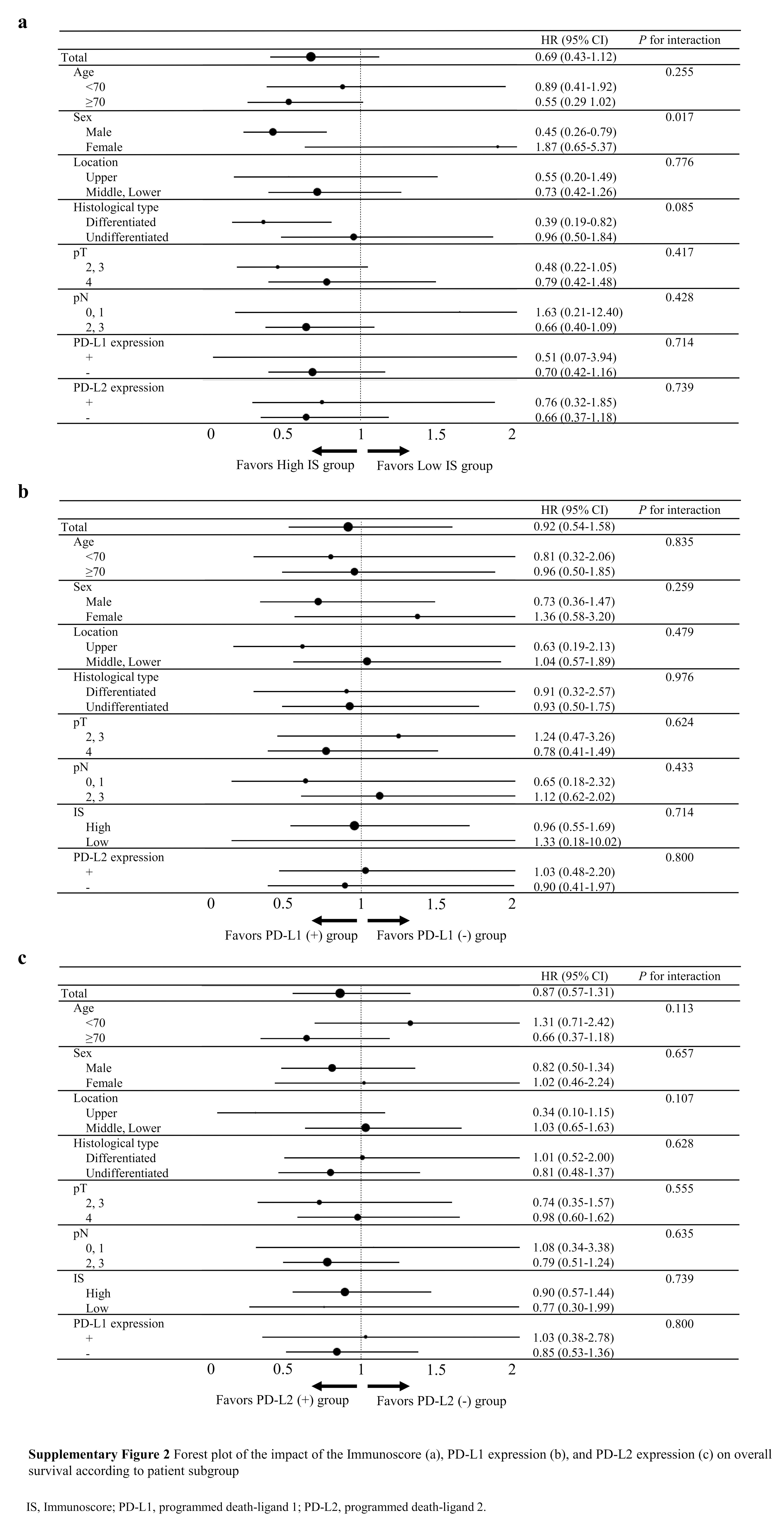

Supplement: Supplementary file 2 — Figure S2: Forest plot of the impact of (a) Immunoscore, (b) PD‐L1 expression, and (c) PD‐L2 expression on overall survival according to patient subgroup. [file AGS3-10-431-s005.tif]

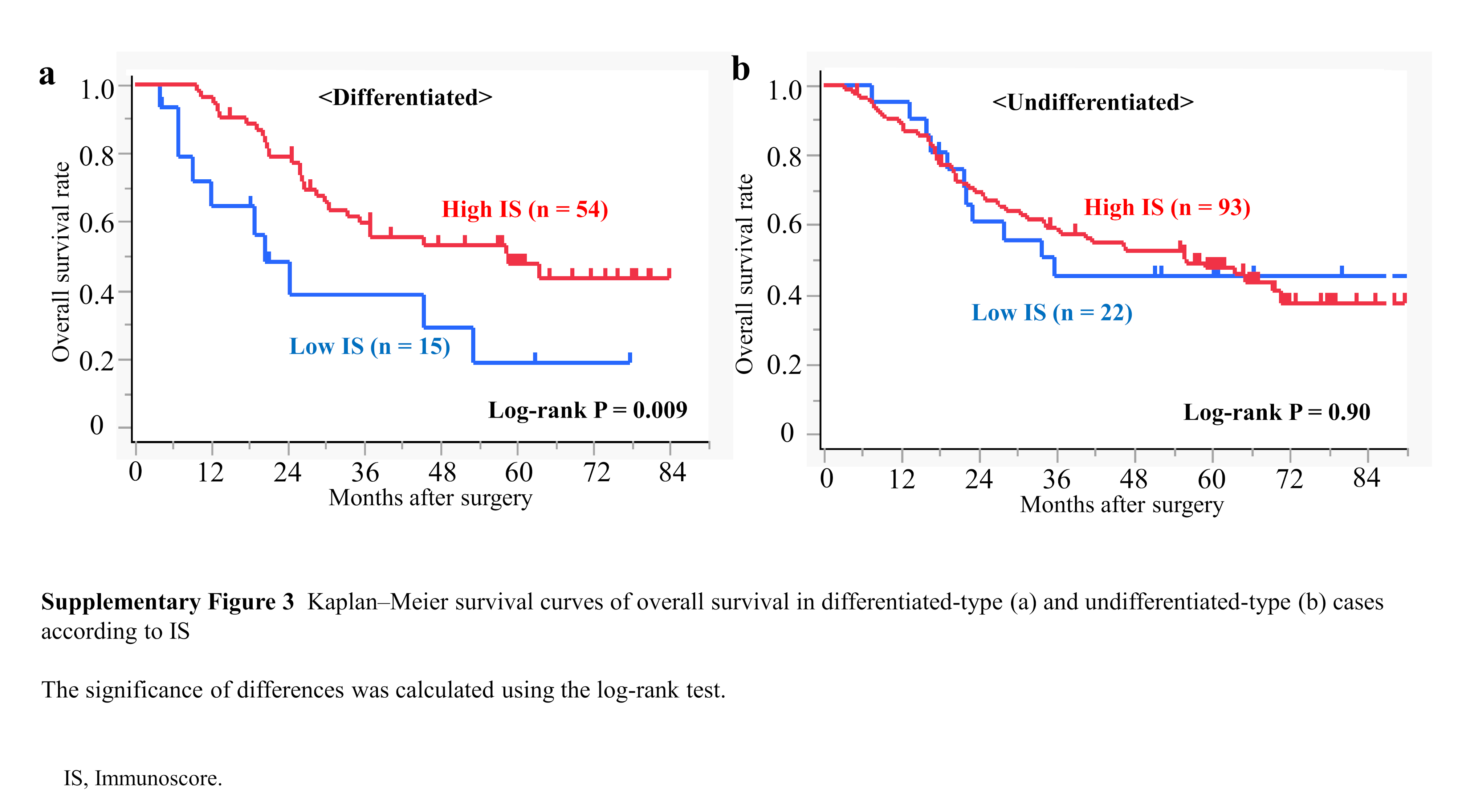

Supplement: Supplementary file 3 — Figure S3: Kaplan–Meier survival curves of overall survival in (a) differentiated‐type and (b) undifferentiated‐type cases according to IS. The significance of differences was calculated using the log‐rank test. [file AGS3-10-431-s002.tif]
