# Supplementary material for: Prognostic Impact of Immunoscore in Pathological Stage III Differentiated Gastric Cancer: A Multicenter Cohort Study Including PD‐L1/PD‐L2 Expression Analysis
Source: Ann Gastroenterol Surg. 2025 Oct 29;10(2):431–42. doi: 10.1002/ags3.70114 (PMC12962046; doi:10.1002/ags3.70114)
Supplement: Supplementary file 5 — Table S1: ags370114‐sup‐0005‐TableS1.docx. [file AGS3-10-431-s003.docx]

**Supplementary Table 1** Clinicopathological characteristics according to histological type

|  | Differentiated  (n = 69) | Undifferentiated  (n = 115) | *P* |
| --- | --- | --- | --- |
| Age |  |  | 0.22 |
| <70 | 26 (38%) | 54 (47%) |  |
| ≥70 | 43 (62%) | 61 (53%) |  |
| Sex |  |  | < 0.001 |
| Male | 59 (86%) | 72 (63%) |  |
| Female | 10 (14%) | 43 (37%) |  |
| Location |  |  | 0.82 |
| Upper | 14 (20%) | 25 (22%) |  |
| Middle or Lower | 55 (80%) | 90 (78%) |  |
| pT |  |  | 0.018 |
| 2, 3 | 31 (45%) | 32 (28%) |  |
| 4 | 38 (55%) | 83 (72%) |  |
| pN |  |  | 0.57 |
| 0, 1 | 12 (17%) | 24 (21%) |  |
| 2, 3 | 57 (83%) | 91 (79%) |  |
| IS |  |  | 0.67 |
| High | 54 (78%) | 93 (81%) |  |
| Low | 15 (22%) | 22 (19%) |  |
| PD-L1 expression |  |  | 0.14 |
| (+) | 8 (12%) | 23 (20%) |  |
| (-) | 61 (88%) | 92 (80%) |  |
| PD-L2 expression |  |  | 0.91 |
| (+) | 24 (35%) | 41 (36%) |  |
| (-) | 45 (65%) | 74 (64%) |  |

IS, Immunoscore; PD-L1, programmed death-ligand 1; PD-L2, programmed death-ligand 2.

**Supplementary Table 2** Univariate and multivariate analyses for overall survival in differentiated-type **(a)** and undifferentiated-type **(b)** cases

**(a)**

| Variables | Category | Univariate analysis | |  | Multivariate analysis | |
| --- | --- | --- | --- | --- | --- | --- |
|  |  | HR (95% CI) | *P* |  | HR (95% CI) | *P* |
| Age (years) | ≥70 | 1.51 (0.76-3.01) | 0.24 |  |  |  |
| Sex | Female | 1.61 (0.67-3.88) | 0.29 |  |  |  |
| Location | M/L | 1.16 (0.53-2.55) | 0.71 |  |  |  |
| pT | 4 | 1.44 (0.75-2.76) | 0.28 |  |  |  |
| pN | 2, 3 | 1.89 (0.67-5.33) | 0.23 |  |  |  |
| Adjuvant chemotherapy | No | 1.97 (1.03-3.77) | 0.042 |  | 1.90 (0.99-3.64) | 0.054 |
| IS | Low | 2.55 (1.23-5.29) | 0.012 |  | 2.45 (1.18-5.09) | 0.017 |
| PD-L1 expression | (-) | 1.01 (0.39-3.11) | 0.86 |  |  |  |
| PD-L2 expression | (+) | 1.01 (0.50-1.94) | 0.97 |  |  |  |

**(b)**

| Variables | Category | Univariate analysis | |  | Multivariate analysis | |
| --- | --- | --- | --- | --- | --- | --- |
|  |  | HR (95% CI) | *P* |  | HR (95% CI) | *P* |
| Age (years) | ≥70 | 1.05 (0.64-1.72) | 0.86 |  |  |  |
| Sex | Male | 1.04 (0.62-1.75) | 0.87 |  |  |  |
| Location | U | 1.12 (0.63-2.00) | 0.70 |  |  |  |
| pT | 4 | 1.77 (0.96-3.26) | 0.069 |  | 1.77 (0.96-3.27) | 0.068 |
| pN | 2, 3 | 1.46 (0.76-2.81) | 0.25 |  |  |  |
| Adjuvant chemotherapy | No | 2.35 (1.42-3.88) | <0.001 |  | 2.35 (1.42-3.90) | <0.001 |
| IS | Low | 1.04 (0.54-2.00) | 0.90 |  |  |  |
| PD-L1 expression | (-) | 1.07 (0.57-2.02) | 0.83 |  |  |  |
| PD-L2 expression | (-) | 1.24 (0.73-2.09) | 0.43 |  |  |  |

HR, hazard ratio; M/L, middle or lower third of the stomach; IS, Immunoscore; PD-L1, programmed death-ligand 1; PD-L2, programmed death-ligand 2; U, upper third of the stomach.
